# Dr. Trotter, on Ipecacuanha

**Published:** 1810-07

**Authors:** T. Trotter

**Affiliations:** Newcastle


### *To the Editors of the Medical and Physical Journal.*

GENTLEMEN,

IN page 447, of your last Number, your learned and ingenious Correspondent, Mr. Royston, is mistaken in thinking *his reference*, on the peculiar property of ipecacuanha, the the first on that subject. In p. 183, of my "VIEW OF THE NERVOUS TEMPERAMENT," when narrating the symptoms, I have introduced the following fact. "The true asthma, is, perhaps, never met with, but in constitutions of great nervous irritability. Such to a certainty must be that species of it, which is induced by particular smells. I knew a lady, who was always seized with asthma, whenever rad. ipecac. was pounding in the shop; so sensible was she of this effect, that it was in vain to conceal from her what was going on in the mortar." (2d Ed.)

This occurred about thirty years ago, in the lady of the physician to whom I was first a pupil; and I was twice the innocent cause of the complaint myself. I thought by her being in a remote part of the house, she could not be affected; but it was almost immediately felt; and the paroxysms lasted many hours. This lady was exquisitely nervous.

I have been informed of different cases almost similar: they were all women; but conceiving the observation to be a common one, I did not note them.

I am, &c.

T. TROTTER, M.D.

*Newcastle, June* 12, 1810.

